# Effects of adding corn steep liquor on bacterial community composition and carbon and nitrogen transformation during spent mushroom substrate composting

**DOI:** 10.1186/s12866-023-02894-x

**Published:** 2023-05-27

**Authors:** Ning Sun, Bowen Fan, Fengjun Yang, Liqin Zhao, Mengmeng Wang

**Affiliations:** College of Horticulture and Landscape Architecture, Heilongjiang Bayi Agricultural University, Daqing, 163319 China

**Keywords:** Spent mushroom substrate, Corn steep liquor, Compost, Bacterial community composition, Carbon and nitrogen, Conversion

## Abstract

**Background:**

Carbon and nitrogen are essential energy and nutrient substances in the composting process. Corn steep liquor (CSL) is rich in soluble carbon and nitrogen nutrients and active substances and is widely used in the biological industry. Nonetheless, limited research has been done on the effect of CSL on composting. This work firstly reveals the effect of adding CSL to bacterial community composition and carbon and nitrogen conversion during composting. This study provides the choice of auxiliary materials for the spent mushroom substrate compost (SMS) and some novel knowledge about the effect of bacterial community on C and N cycling during composting of SMS and CSL. Two treatments were set up in the experiment: 100% spent mushroom substrate (SMS) as CK and SMS + 0.5% CSL (v/v) as CP.

**Results:**

The results showed that the addition of CSL enhanced the initial carbon and nitrogen content of the compost, altered the bacterial community structure, and increased the bacterial diversity and relative abundance, which might be beneficial to the conversion and retention of carbon and nitrogen in the composting process. In this paper, network analysis was used to screen the core bacteria involved in carbon and nitrogen conversion. In the CP network, the core bacteria were divided into two categories, synthesizing and degrading bacteria, and there were more synthesizing bacteria than degrading bacteria, so the degradation and synthesis of organic matter were carried out simultaneously, while only degrading bacteria were found in the CK network. Functional prediction by Faprotax identified 53 groups of functional bacteria, among which 20 (76.68% abundance) and 14 (13.15% abundance) groups of functional bacteria were related to carbon and nitrogen conversion, respectively. Adding CSL stimulated the compensatory effect of core and functional bacteria, enhanced the carbon and nitrogen transformation ability, stimulated the activity of low-abundance bacteria, and reduced the competitive relationship between the bacterial groups. This may be why the addition of CSL accelerated the organic matter degradation and increased carbon and nitrogen preservation.

**Conclusions:**

These findings indicate that the addition of CSL promoted the cycling and preservation of carbon and nitrogen in the SMS composts, and the addition of CSL to the compost may be an effective way to dispose of agricultural waste.

**Supplementary Information:**

The online version contains supplementary material available at 10.1186/s12866-023-02894-x.

## Introduction

With an annual production of edible fungi exceeding 13 million tons, China is the largest producer of edible fungi in the world. It accounts for about 75% of the world's total edible fungi production [1]. Nonetheless, large amounts of SMS are not effectively used and are freely discarded, causing environmental and groundwater pollution. Soil application is an ideal method for sustainable utilization of SMS, owing to its low bulk density and well-developed porosity and containing a large number of nutrients (inorganic nitrogen, phosphorus, potassium, etc.) and organic matter [2]. SMS can slowly and continuously provide nutrients for crops after application in the soil, promote the formation of soil aggregates, improve the physicochemical properties of the soil, promote soil nutrient activation, and improve the activity of aerobic microorganisms [3]. The SMS contains many hard-to-degrade compounds, such as lignocellulose, fat, wax, tannin, etc., and some phytopathogenic bacteria, all of which can produce phytotoxic compounds after degradation, including polyphenols, organic acids, quinones, aldehydes, alcohols, and so on. Therefore, fresh SMS should not be applied directly and must be composted before being used as a fertilizer or as a medium for the cultivation of seedlings [4]. In light of the complex physical and chemical composition of lignocellulose, it degrades slowly, and the high concentration of lignocellulose in SMS frequently results in long composting times, low fermentation temperatures, and incomplete decomposition [2]. Adding some exogenous substances such as inoculating microorganisms [5], bulking agents (biochar, diatomite, etc.) [6, 7], oxidants (MnO_2_, etc.) [8] and molasses amendment [9] can accelerate the aerobic composting process and improve the quality of compost, which is an efficient method for treating SMS.

Composting is primarily driven by microorganisms, primarily bacterial communities, which decompose organic matter and form new humic compounds through secondary metabolism [10–12]. The impact of microorganisms on composting depends on the structure and functional capacity of the microbial community [11, 13]. Microbial community succession has a direct influence on the degradation process in the composting process, which is also the key factor to promote the degradation of organic matter [14, 15]. Moreover, carbon and nitrogen conversion is the most important biochemical process in composting [16]. Sugars, cellulose, and lignin can be hydrolyzed by microorganisms secreting hydrolytic enzymes under good aeration conditions, and the end products are CO_2_, H_2_O, and inorganic salts. While under anaerobic conditions, organic acids and reducing gases (CH_4_, H_2_, etc.) are formed. Under the action of microorganisms, nitrogenous organic compounds degrade to produce amino acids, polyphenols, peptides, organic acids, alcohols, and other intermediates and inorganic nitrogen (NH_4_^+^, NO_3_^−^) by hydrolysis, ammonification, nitrification, and denitrification. By oxidizing polyphenols to quinones by microbial oxidase, these intermediates serve as precursors for humic acid synthesis, and then humic acid is produced by condensing the quinones with amino acids and peptides, a stable compound able to preserve carbon and nitrogen in compost [12]. During the composting process, releasing CO_2_ and CH_4_ gases causes carbon nutrient losses; nitrification, denitrification, and volatilization of ammonium lead to nitrogen losses. Severe carbon and nitrogen losses significantly affect microbial activity and reduce the conversion rate of organic matter and compost product quality. Furthermore, the released greenhouse gases (N_2_O, CO_2_, and CH_4_, etc.), in turn, cause pollution to the environment [17]. Therefore, in order to accelerate composting, enhance compost quality, and protect the environment, it is essential to study carbon and nitrogen cycles and reduce greenhouse gas emissions.

Corn steep liquor (CSL), a by-product in the industrial wet processing of corn starch, is a dark brown liquid after concentration [18]. In addition to soluble sugars, soluble proteins, organic acids, vitamins, enzymes, and inorganic ions, CSL is an excellent source of nitrogen and carbon for microbial fermentation [19]. Moreover, CSL is widely utilized in many fields due to its high yield, low price, and abundant supply [18]. For example, it is used for microbial culture medium [20], degradation of organic pollutants [21], a raw material for biological agents [22], and plant disease control [23]. However, the application of CSL in composting has not been reported.

Our previous study demonstrated that adding 0.5% CSL could promote carbon and nitrogen metabolism during the preparation of *Stropharia* substrates, and the abundant soluble sugars and proteins in CSL provided energy and an effective nitrogen source for the mass reproduction of microorganisms. In this study, SMS (*A. bisporus*) was used as experimental material to study the biological mechanism of bacterial-driven carbon and nitrogen transformation in composting by adding CSL. This study aims to (1) investigate the effects of adding CSL on the bacterial community structure and composition during SMS composting; (2) identify the core bacterium involved in carbon and nitrogen transformation; (3) evaluate the effects of functional bacteria on the transformation of carbon and nitrogen; (4) explore the potential mechanism of core and functional bacteria involved in carbon and nitrogen transformation in SMS composting.

## Material and methods

### Composting experimental setup

​SMS (*A. bisporus*) was obtained from the Hengrui Edible Mushroom Cooperative in Datong District, Daqing City, Heilongjiang Province, and CSL was obtained from the College of Food, Heilongjiang Bayi Agricultural University. The experiment was conducted in August 2021, and the composting was carried out for 61 days at the experimental site of Heilongjiang Bayi Agricultural University, China. And the experiment was conducted with two treatments: CK treatment was pure SMS; CP treatment was SMS with 0.5% CSL (fresh sample v/v), uniformly mixed and subsequently fermented in a conical pile of 2.5 m × 1.5 m × 1.5 m (length × width × height) under natural conditions. The initial moisture content of the compost was about 60%, and the stack and ambient temperature were measured daily. The physical and chemical properties of the raw materials were displayed in Table 1, and the stack was manually turned three times (10th d, 20th d, 29th d) when the temperature was cooled down.Compost is considered to have reached maturity standards when the temperature is near room temperature and does not rise.

**Table 1:** Characteristics of raw materials before composting

Name	pH	EC/(mS/cm)	TOC/(g/kg)	TN/(g/kg)	TP/(g/kg)	TK/(g/kg)
SMS	8.013	3.31	206.05	15.34	9.35	10.21
CSL	-	-	85.85	238.00	12.53	10.29

*SMS* Spent mushroom substrate, *CSL* Corn steep liquor, *EC* Electrical conductivity, *TOC* Total organic Carbon, *TN* Total nitrogen, *TP* Total phosphorus (P_2_O_5_), *TK* Total potassium (K_2_O)

### Sample collection and determination of the physicochemical properties

Samples were collected from the pile on the 1st, 3rd, 5th, 10th, 17th, 29th, 36th, and 61st d. Moreover, samples were taken from 20 cm, 70 cm, and 120 cm away from the pile top, and three samples taken at each depth were mixed to make subsamples. The subsamples were divided into two parts: one was stored at -80℃ for nucleic acid extraction, and the other was air-dried and crushed to determine physicochemical indexes. Based on temperature change, the collected samples from 1st, 3rd, 17th, and 36th d were picked to represent mesophilic, thermophilic, cooling, and maturation stages, respectively, for high throughput sequencing.

In this experiment, the stack and ambient temperatures were measured using a Maxon DS192X automatic temperature recorder with memory storage function; water content (WC) was determined by drying the samples to constant weight in a 105℃ oven; pH and EC were determined by a pH meter and a conductivity meter; total organic carbon (TOC) was determined via an externally-heated potassium dichromate-volumetric method; total nitrogen (TN) content was determined by Kjeldahl method; ammonium nitrogen and nitrate nitrogen were extracted by 2 M KCl solution and determined by spectrophotometry.

### DNA extraction and high-throughput 16S rRNA pyrosequencing

Composting samples (3 ~ 5 g) stored at -80℃ were analyzed for 16S rRNA library construction and sequencing at Beijing Allwegene Technology Co., Ltd., with the amplification region of V3-V4 and primers of 338 F and 806 R. Subsequently, the extracted DNA was separated by 1% agarose gel electrophoresis to test its quality and then amplified by PCR, following the purification with the Agencourt AMPure XP Kit (Beckman Coulter, Inc., USA). Subsequently, the libraries were constructed and sequenced by the MiseqPE300 platform (Beijing, Allwegene), and the obtained raw sequence data of 16S ribosomal RNA (16S rRNA) were used for subsequent bioinformatics analysis. The clean_tags were obtained after removing chimeras and short reads from the original sequences by the Uchime method and clustered into operational taxonomic units (ASVs) at a 97% threshold. Alpha and Beta diversity analyses were performed based on the clustering results, and the taxonomic information of species at each level was obtained by the annotation results.

### Statistical analysis

SPSS 26.0 was used for the statistical analysis of the data in this experiment, and TOC and TN data were analyzed and plotted by Origin 2020. Network analysis was used to identify the core bacteria involved in carbon and nitrogen conversion during composting [24]. Bacterial diversity indices and OTU abundance were calculated by the "vegan" package in R (https://www.r-project.org/). Alpha diversity index (including ACE index and Shannon index) and PCoA plot ImageGP using online tools (http://www.ehbio.com/ImageGP/).Pearson correlations between microbial taxa and physicochemical parameters were performed by the "Hmisc" package of R (| r |> 0.7; *p* < 0.05). Pearson correlations between bacterial genus and physicochemical parameters were performed by the "Hmisc" package of R (| r |> 0.7; *p* < 0.05). In addition, Faprotax database was used for bacterial function prediction, and based on the prediction results, functional bacterial populations involved in carbon and nitrogen transitions were identified. The co-occurrence network data was processed using the "plot (g)" package, and Gephi software was used to edit and calculate the topological properties visually. Subsequently, structural equation modeling (SEM) was performed using AMOS software (IBM; SPSS AMOS 20.0.0) to assess the effect of core and functional bacteria on carbon and nitrogen transformation.

### Availability of data and materials

All data generated or analyzed during this study are included in the article. Also, all the raw sequences were deposited in the NCBI sequence read archive (SAR) under BioProiect PRJNA935335, http://www.ncbi.nlm.nih.gov/bioproject/935335.

## Results and discussion

### The change of TOC and TN during composting

**Fig. 1:**
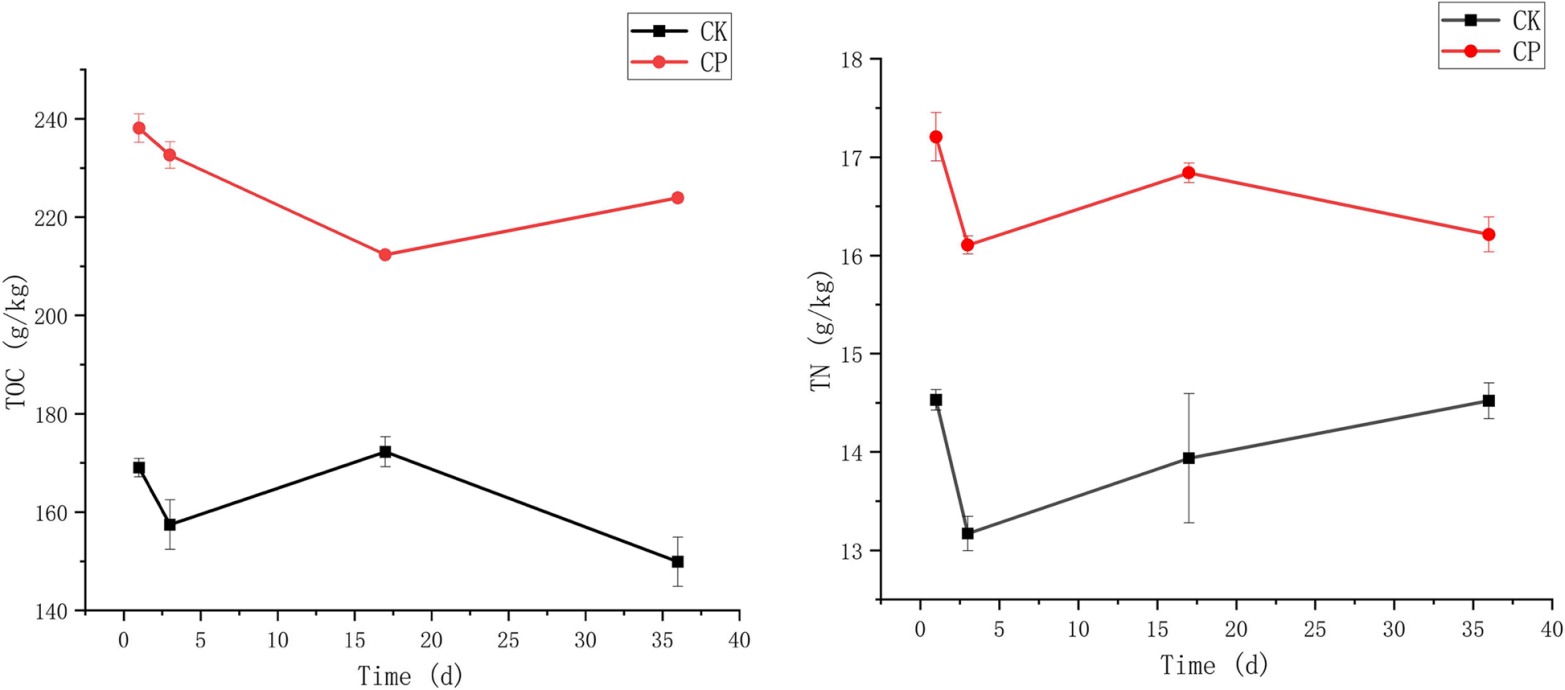
Changes in total organic carbon (TOC) and total nitrogen (TN) during composting. It can be seen from the figure that although the changing trends of the two treatments are not the same, the TOC and TN of the CP treatment are always higher than CK treatment in each period

​Carbon and nitrogen metabolism is the most important metabolic process in composting, and the carbon and nitrogen content is also an essential evaluation criterion for the quality and maturity of compost. The changes in TOC and TN during composting are shown in Fig. 1. The content of TOC exhibited decreasing trends in both treatments, but the changing trend was different, which might be caused by consuming a large amount of organic carbon by microbial activity. The TOC content in CK and CP composts decreased from 169.04 g/kg and 238.09 g/kg to 149.90 g/kg and 223.91 g/kg, respectively, with a decrease of 11.32% and 5.96%, indicating that adding CSL could effectively reduce the carbon loss during SMS composting. This may be related to the increased initial carbon and nitrogen content in compost material by adding CSL (Table 1). The TOC content persistently decreased until an increase to colling period, and it was higher at maturation stages than that at colling period. While the TOC content in CK compost decreased rapidly from the mesophilic period to the thermophilic period, increased during the cooling period, and then decreased rapidly again. This may be related to the amount of available organic carbon in the two treatments. The CK compost had less available carbon in the initial period, microbial reproduction consumed a large amount of effective carbon source in SMS, and the CP treatment increased the initial available organic carbon due to the addition of CSL (Table 1), reducing the consumption of organic carbon in SMS. In addition, many bacteria (*Proteobacteria*, *Firmicutes*, *Chloroflexi*, *Chlorobia*, *Actinobacteria*, *Thermodesulfobacteria*, etc.) and archaea (*Euryarchaeota*, *Crenarchaeota*) can immobilize CO_2_ [25], and the microbial biofixation of carbon leads to an increase in TOC content [26]. The overall trends of TN in the CK and CP treatments were similar, with a rapid decrease at the beginning of composting and then an increase. The rapid decrease in TN content at the beginning of composting may be due to the NH_3_ volatilization due to NH_4_^+^ produced during the mineralization of organic matter, while the increase in TN content of compost caused by biological nitrogen fixation through microorganisms [26] and the Faprotax functional prediction (Fig. 5B) also supports this inference. Bacteria are the main nitrogen-fixing microorganisms [27, 28], and there are six microbial nitrogen fixation pathways, including three aerobic (the Calvin cycle, the 3-hydroxypropionate cycle, and the 3-hydroxypropionate-4-hydroxybutyrateaq cycle) and three anaerobic nitrogen fixation pathways (the reductive TCA cycle; dicarboxylate/4-hydroxybutyrate cycle and wood-Ljungdahl pathway) [29]. Meng et al. have observed that adding molasses amendment to SMS compost can promote organic matter degradation and humification, reducing NH_3_ volatilization and N_2_O release [9]. Abundant soluble sugars and proteins in CSL, were with the same effect as molasses amendment, providing plenty of energy and nutrients for microbial activities during composting.

### Changes in the bacterial community composition during co-composting

**Fig. 2:**
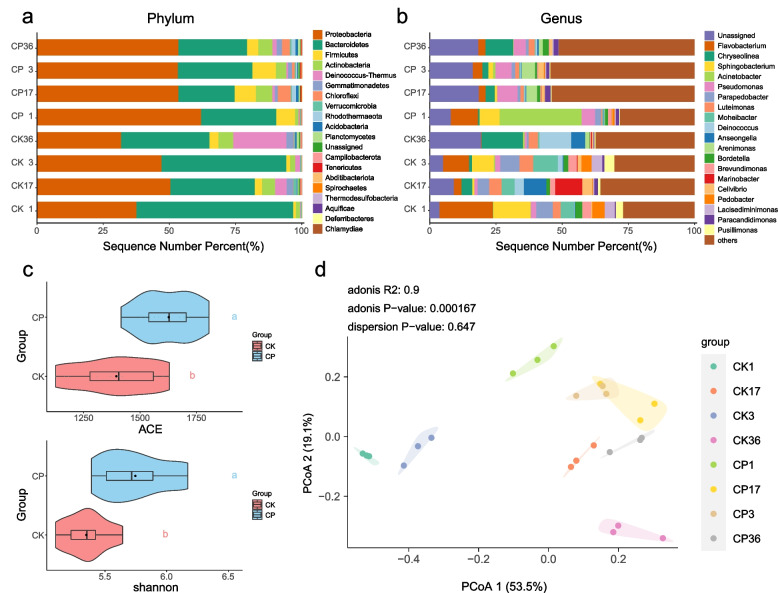
Variations of bacterial community during composting. The top 20 phyla (**A**) and genus (**B**) relative abundance; Alpha diversity indices of the bacterial community in composting (**C**); Principal coordinates analysis of the bacterial community in composting (**D**). It can be seen from the figure that there is a great difference in microbial composition between CP and CK treatment at the gate level and genus level. The problems of fast degradation of organic matter and high reactor temperature can be explained through microbial function. Alpha diversity and PCOA could further explain that adding corn pulp increased microbial community diversity and changed microbial community structure

The bacteria at the top 20 phyla and genera were shown in Fig. 2. The dominant phyla of compost were *Proteobacteri* (31.57%-61.80%) and *Bacteroidetes* (21.23%-59.23%); and *Firmicutes* (0.99%-8.80%), *Actinobacteria* (1.27%-6.21%) and *Deinococcus-Thermus* (0.24%-20.13%) were widely present in different composting stages (Fig. 2A). In CK composting, *Proteobacteria* showed an increasing first and then decreasing trend, with the highest abundance during the cooling period (50.27%); In CP composting, the relative abundance was the highest in the period of mesophilic (61.80%) and then decreasing, but the CP treatment was always higher than the CK treatment. *Bacteroidetes* indicated a higher abundance at the beginning of composting, and then a decrease in both treatments, and the lowest abundance was observed in the cooling period. The addition of CSL increased the relative abundance of *Firmicutes* and *Actinobacteria*, notably during the mesophilic, thermophilic, and cooling phases. *Proteobacteria* [30], *Firmicutes* [31], *Actinobacteria* [32], and *Bacteroidetes* [33] are widely recognized as important phyla for lignocellulose degradation and play key roles in carbon and nitrogen conversion [34]. The increased abundance of *Proteobacteria*, *Firmicutes*, and *Actinobacteria* after adding CSL may be one of the reasons for the faster organic matter degradation and higher pile temperature in the CP treatment (Fig. S1). This is consistent with the functional prediction (Fig. 5) that adding CSL could enhance the capacities of microbial carbon sequestration and organic carbon degradation.

At the genus level, there was some difference in the bacterial community composition between the CK and CP (Fig. 2B). The dominant genera in both treatments were *Flavobacterium* (6.53%), *Chryseolinea* (4.70%), *Acinetobacter* (4.13%), *Sphingobacterium* (4.20%), *Pseudomonas* (3.70%), *Parapedobacter* (3.06%), *Deinococcus* (2.28%). The relative abundance of *Bordetella*, *Pedobacter*, *Paracandidimonas*, *Lysobacter*, and *Pusillimonas* was between 1–2%. *Acinetobacter* (*Bacillus spp.*) had the highest abundance (30.97%) at the beginning of CP composting, and *Acinetobacter*, which form spores under adverse conditions such as high temperature and drought, has a good effect on the decomposition of organic matter and promote carbon and nitrogen metabolism [35]. *Chryseolinea* showed a gradual increase from the beginning of composting in both the CK and CP, while *Flavobacterium* and *Sphingobacterium* gradually decreased. The abundance of *Pseudomonas* in the CP compost was significantly higher than that in the CK treatment, and the highest abundance was found in the thermophilic and cooling periods. Previous studies found that *Flavobacterium*, *Pseudomonas*, *Sphingobacterium*, and *Pedobacter* were involved in lignocellulose degradation by secreting lignocellulose degrading enzymes [36–38]. *Pseudomonas* can also promote the humic acid formation, increasing the degree of polycondensation, aromatization, and molecular size, forming macromolecular humus, and further facilitating carbon sequestration [37]. This may be due to the high content of soluble sugars in CSL (Table 1), which promotes the compost humification process [9] and reduces carbon loss during composting. *Pseudomonas* is also involved in denitrification under aerobic conditions [33, 39]. These results precisely explain the decrease in TOC and increase in TN in the CP compost during the cooling period. *Deinococcus* (a NapA-type denitrifying bacterium) [39] was significantly more abundant in CK compost than in CP treatment and increased continuously during the composting, and relative abundance reached 12.00% in the maturation period, which was associated with a significant increase in NO_3_^−^ content in the late stage of composting in CK treatment (Figure S2). *Pusillimonas* can produce carboxymethylcellulose, which is involved in the degradation of polysaccharides and lignocellulose [40]. Yet, high temperature inhibits its growth [41]. The temperature of CP is consistently greater than that of CK, resulting in a consistently higher abundance of *Pusillimonas* in the CK treatment than in CP.

ACE and Shannon indices were used to assess bacterial communities' abundance and diversity in this experiment. ACE and Shannon indices (Fig. 2C) indicated that the addition of CSL significantly increased bacterial communities' diversity and abundance, and the higher the diversity and abundance of the bacterial community, the greater the contribution to the conversion of organic components [42]. Additionally, PCoA was used to further elucidate the changes in bacterial community structure during the CP and CK composting. PCoA results showed (Fig. 2D) that the bacterial community structure changed significantly at different composting stages. The addition of CSL caused the Bray–Curtis distance matrix of the bacterial community structure to be closer than CK, indicating that the bacterial community composition was similar in different composting stages. The closer distance matrix of the microbial community structure indicates a higher complexity of microbial interactions [8]. Overall, adding CSL altered the bacterial community structure by increasing its diversity and richness, further facilitating the compost's decomposition and organic matter conversion.

### Identifying core bacteria in the carbon and nitrogen transformation and interspecies relationships during composting

**Fig. 3:**
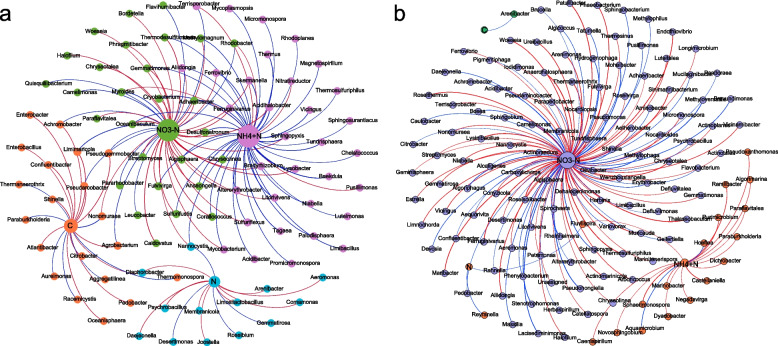
Network analysis of organic carbon and nitrogen transformation and related bacterial genera according to Pearson correlation analysis (*p* < 0.05) in CP (**A**) and CK (**B**)composting. The red line is a positive correlation, and the blue line is a negative correlation. The node is a genus. As can be seen from the figure, the addition of corn pulp promoted more bacteria to participate in carbon and nitrogen metabolism, improved the connectivity between bacterial communities, and stimulated low-abundance bacteria to participate in carbon and nitrogen conversion

It is important to note that the conversion of carbon and nitrogen in composting is not only influenced by the composition of the microbial community but is also closely related to the functional capacity of the microbial community [11, 13]. In this study, network analysis was used to identify the core bacteria at the genus level involved in the carbon and nitrogen transformation during composting, screening the bacteria, which significantly correlated with the transformation of TOC and TN (*p* < 0.05), and constructing network analysis models. The red and blue lines in the figure represent a significant positive and negative correlation, respectively, and each node is a genus. In the CP network, 14 positively- and 7 negatively-correlated nodes were involved in the TOC conversion (positive correlation 66.67%), and 9 positively- and 4 negatively-correlated nodes were involved in the TN conversion (positive correlation 69.23%) (Fig. 3A). 2 and 1 negatively-correlated nodes were related to the TOC and TN conversion in the CK network, respectively (Fig. 3B), indicating that adding CSL promoted more bacterial genera to participate in carbon and nitrogen metabolism. The core bacterial genera were divided into two categories: syntrophic and degradative bacteria, and there were more syntrophic bacteria than degradative bacteria, while only synthetic bacteria were present, and very few species were in the CK network. This may be one of the reasons for the faster degradation of organic matter and less loss of nutrients in CP composting. There were 17 (88.24% positive correlation) and 49 (10.21% positive correlation) core bacterial genera associated with NH_4_^+^, and 118 (59.32% positive correlation) and 48 (72.92% positive correlation) core bacterial genera associated with NO_3_^−^ in the CK and CP networks, respectively, indicating that the addition of CSL reduced the core bacterial genera associated with NO_3_^−^ bacteria, while the increase of NH_4_^+^-related core bacterial genera, this was beneficial to inhibit NO_3_^−^ formation and promote the conversion of NO_3_^−^ to NH_4_^+^. This was consistent with the functional prediction (Fig. 5). Yunxian Zhang et al. found that the inoculation of functional microbial agents could significantly increase the content of organic nitrogen, while decease the mineralization rate of nitrogen [43]. The ammonium nitrogen content at high temperature can affect the ammonia emission, thereby facilitating nitrogen fixation [44]. This is consistent with the conclusion that adding corn steep liquor has changed the microbial community structure of the pile, and thus increasing the total nitrogen content. According to María del Carmen Muñoz-Marín et al., it was verified that microorganisms could reduce N_2_ in the atmosphere to bioavailable ammonium nitrogen, which could provide inorganic nitrogen for ecosystems [45]. In this study, the core genus of ammonium nitrogen disposed by CSL was more than that in CK treatment. Moreover, the total nitrogen content of the former was higher than that of the latter, which might result from more core bacteria associated with N_2_ fixation in the CSL treatment. This was also intrinsic to the higher total nitrogen content in the CSL treatment [46–48].

*Arenibacter* (an algicidal bacteria) participated in converting TOC and TN in the CK and CP networks, suggesting that *Arenibacter* is involved in both carbon and nitrogen metabolism. Eight and one bacterial genera in the TOC module of the CP network were present in the nitrate and ammonia nitrogen modules of the CK network, suggesting that environmental factors influence the function of core bacteria. As a whole, adding CSL could significantly increase the Hub bacteria among the modules, and the network's connectivity was improved by connecting all modules through Hub bacteria. In addition, the CP and CK networks also consisted of 131 and 137 core bacterial genera, corresponding to 6 and 12 of the top 20 genera, respectively, indicating that the efficiency of the top 20 genera decreased after adding CSL. In contrast, the activity of the core genera with lower relative abundance was stimulated and promoted carbon and nitrogen conversion during composting. Similarly, Qi et al. concluded the same by adding MnO_2_ to the compost [12]. This may be related to the amount of carbon and nitrogen nutrients and active substances in the CSL.In addition, genera with lower relative abundance and genera with higher relative abundance had specific functions, with the former being more inclined to cooperate than the latter [49–51]. After adding CSL, the genus with lower relative abundance could play a greater role as the core genus. This might be a reason for promoting the transformation of carbon and nitrogen.

**Fig. 4:**
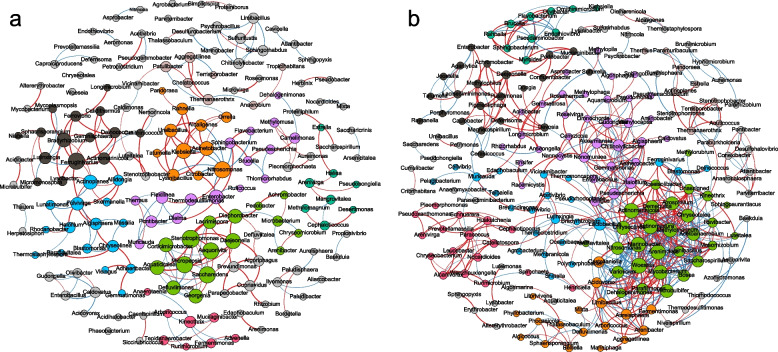
Co-occurrence network diagram at bacterial genus level in CP (**A**) and CK (**B**) composting. Co-occurrence network diagrams are colored by the bacterial phyla and key taxa, respectively. The red line is a positive correlation, and the blue line is a negative correlation. The node is a genus, and the width of the line means the degree of correlation. It can be seen from the figure that the addition of corn pulp improves the connectivity between nodes and reduces the complexity of the network diagram. Meanwhile, it is found that the positive correlation rate among the flora increases after the addition of corn pulp, which promotes the complementarity effect of the flora, which may be related to the transformation of carbon and nitrogen

A co-occurrence network (Fig. 4) was adopted to explore the effect of adding CSL on the ecological processes of bacterial communities during co-composting based on a Pearson correlation analysis (*p* < 0.05). The red and blue lines represent a positive and negative correlation, respectively, and the thickness of the line represents the degree of the correlation; each node represents a genus, and the node's size represents the degree of the genus in the co-occurrence network. A positive correlation indicates a symbiotic relationship and a negative correlation indicates a competitive or predatory relationship. As indicated in Fig. 4, topological features in CK and CP co-occurrence networks are significantly different (Table S1). The co-occurrence network of CP (Fig. 4A) consists of 172 nodes, 246 edges, and 158 total triangles, while the co-occurrence network of CK (Fig. 4B) consists of 207 nodes, 625 edges, and 1412 total triangles. The network graph density (0.029) and average degree (6.039) of CK are higher than those of CP (0.017, 2.86). The average clustering coefficients and path lengths of the two networks are not significantly distinct. Yet, the number of connected components of the CP network (28) is significantly higher than that of CK (16), indicating that adding CSL can improve the connectivity of the co-occurrence network and yet reduce the complexity, consistent with the results of the core bacterial network (Fig. 3). At the same time, this study found that the ratio of positive correlations between the bacterial community increased after adding CSL (CP:59.76%; CK:57.44%), positive correlations represented reciprocal or symbiotic relationships. These results showed that adding CSL increased the available carbon and nitrogen sources for microorganisms and activated the complementary effect of the core bacteria, further improving the bacterial metabolic capacity [52]. The complementary effect between microorganisms is to share resources through cross-feeding, which leads to more efficient bacterial growth [53]. The core bacteria involved in carbon and nitrogen transformation were all present in the co-occurrence network, and the complementary effect of core bacteria was stimulated, which may account for the accelerated carbon and nitrogen transformation. In addition to the factors that accelerated the conversion of carbon and nitrogen in the reactor through the complementary effect, the stability of the co-occurrence network tends to decrease while specific functions are enhanced [54, 55]. The enhanced specific function of the microbial community with the addition of CSL might be another reason for the acceleration of carbon and nitrogen conversion in the pile.

### Predicted bacterial function during composting

**Fig. 5:**
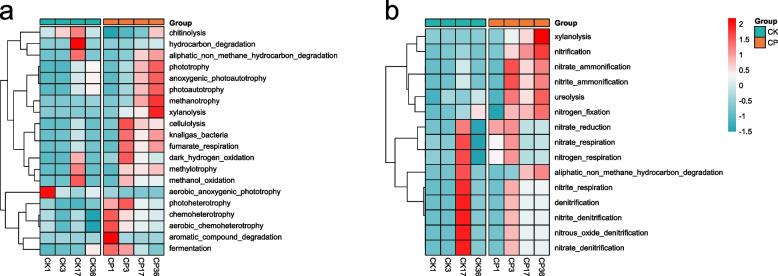
Prediction of functional bacterium of CP and CK compost at different time points. Functional bacteria of carbon (**A**) and nitrogen (**B**) transformation. According to the functional prediction, the functional flora related to the conversion of carbon and nitrogen increased significantly after the addition of corn pulp, and the metabolic capacity of carbon and nitrogen was enhanced. The changes in TOC and TN in the composting process could be perfectly explained through the analysis of the heat map

In this study, 16S high-throughput sequencing data and Faprotax software were adopted to predict the functions of bacterial community during the SMS composting (Fig. 5). A total of 53 functional groups of 1002 OTUs were obtained by the FAPROTAX tool, of which 20 (76.68% abundance) and 14 (13.15% abundance) groups of functional bacteria were associated with carbon and nitrogen conversion, respectively. Adding CSL significantly increased the abundance of functional bacteria communities involved in carbon and nitrogen conversion and promoted carbon and nitrogen metabolism. In composting, carbon and nitrogen are converted through synthetic and degradation pathways, and the net rate of these two pathways determines the amount of TOC and TN in compost.

Both autotrophic and heterotrophic microorganisms can fix CO_2_, and autotrophic microorganisms use CO_2_ as the only carbon source to synthesize organic compounds; heterotrophic microorganisms can fix CO_2_ through carboxylation reactions, and the substrate for fixing CO_2_ comes from the degradation of organic compounds [26]. Compared with CK, the addition of CSL improved chemoheterotrophy and aerobic chemoheterotrophy functions in the early stages, and photoautotrophy and anoxygenic photoautotrophy functions in the late stages (Fig. 5A), indicating that adding CSL improves the organic carbon synthesis capacity, promotes the humification process and facilitates the carbon sequestration in compost. Microbial fermentation can release CO_2_ through decarboxylation, redox, and ATP synthesis pathways [26]. Microbial fermentation function was more robust in the CP treatment than in the CK from mesophilic to cooling periods. The peak of aromatic compound degradation appeared in the mesophilic period, degradation of aliphatic non-methane hydrocarbons, methanotrophy, and xylanolysis occurred mainly in the cooling and maturation periods, and dark hydrogen oxidation and cellulolysis degradation peaked in the thermophilic period; methylotrophy and methanol oxidation were always faster after the thermophilic period. Overall, carbon metabolism in CP compost was stronger than that in CK (Fig. 5A). The degradation of these complex organic compounds produced CO_2_ and a large number of intermediate products, which could provide precursors for humic acid formation, suggesting that adding CSL can promote the degradation of organic carbon and humus formation. This may be the reason for the decrease of TOC in the early stage and the rapid increase in the later stage of CP composting, consistent with the findings of Meng et al. [9].

The addition of CSL significantly promoted nitrogen transformation during composting (Fig. 5B). Nitrogen is converted quickly throughout the composting process in CP treatment, while nitrogen transformation was the fastest during the cooling period in CK (Fig. 5B). The addition of CSL improved the functions of ureolysis, nitrogen fixation, nitrification, nitrate, and nitrite ammonification of compost after the thermophilic period. Ureolysis has an important impact on microbial communities [56], and microbial ureolysis can promote the dissolution of precipitated phosphate and improve the availability of phosphorus [57]. Effective phosphorus is the basis for microbial synthesis of phosphorus-containing macromolecules and carbon and nitrogen metabolism. Nitrification, nitrate, and nitrite ammonification promote the interconversion between NO_3_^−^ and NH_4_^+^ and facilitate maintaining TN's balance. Except for the cooling period, nitrate reduction, nitrate respiration, and nitrogen respiration functions of CP were significantly higher than CK. Nitrate reduction could convert nitrate nitrogen to ammonium nitrogen, reduce NO_3_^−^ leaching, and mitigate groundwater pollution [58]. After adding CSL, the nitrogen loss caused by denitrification and respiration mainly occurred during and after thermophilic, while nitrogen loss of CK treatment mainly occurred during the cooling period. After the thermophilic period, the nitrogen fixation ability of bacteria in CP compost was significantly higher than in CK, resulting in higher nitrogen retention in the compost product. Functional predictions can perfectly account for the variations in TOC and TN.

### The impact factors of carbon and nitrogen transformation during composting

**Fig. 6:**
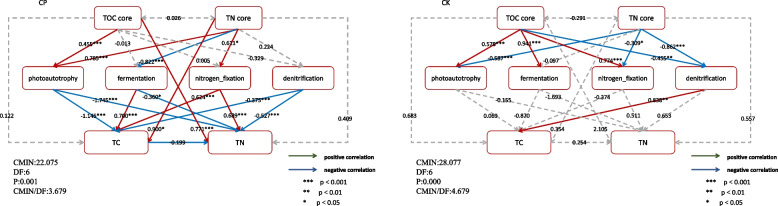
The contribution of core and functional bacteria to transforming carbon and nitrogen. Carbon sequestration and loss represent the data derived from the photoautotrophy and fermentation functional bacteria. * means *p* < 0.05; ** means *p* < 0.01; *** means *p* < 0.001. The red and blue arrows represent the positive and negative relationship, and the gray dotted arrow indicates that the correlation is not significant. According to the structural equation model, the carbon and nitrogen conversion ability of the core and functional bacteria in the composting process was significantly enhanced after the addition of corn pulp, and the carbon and nitrogen cycling ability of the whole process was promoted through the complementary effect

In this study, structural equation modeling (SEM) was used to investigate the effects of core bacterial community and functional bacteria on carbon and nitrogen cycling (Fig. 6), showing that adding CSL could significantly improve the carbon and nitrogen transformation ability of core bacteria and functional bacteria. In the CK composts (Fig. 6), TOC core bacteria were significantly and positively correlated with the carbon-fixing, carbon-consuming, and nitrogen-fixing ability but significantly and negatively correlated with the denitrifying functional bacteria; TN core bacteria were significantly and negatively correlated with the carbon-fixing, nitrogen-fixing, and denitrifying functional bacteria; TOC and TN core bacteria indirectly and significantly affected TOC content through denitrifying functional bacteria, and had no significant effect on TN content.

The addition of CSL significantly enhanced the carbon and nitrogen conversion ability of core and functional bacteria during the composting (Fig. 6). Both core and functional bacteria have direct and significant effects on the TOC and TN content; core bacteria also indirectly influence the transformation of TOC and TN by the functional flora. The TOC and TN core bacteria directly and significantly affected the TN and TOC content, respectively, indicating that adding CSL stimulated the complementary effect of the core bacteria. In addition, TOC core bacteria indirectly regulated TOC and TN conversion in the compost by influencing the carbon sequestration capacity of functional bacteria; TN core bacteria indirectly regulated the carbon and nitrogen conversion through carbon-sequestering, carbon-consuming, and nitrogen-fixing bacteria; denitrifying bacteria had a significant negative correlation with TOC and TN. This stems from the fact that carbon and nitrogen elements affect the metabolic function of their core genus, thus influencing its conversion [59, 60]. These results further indicated that the interaction effect between core and functional bacteria was significantly enhanced by adding CSL, which promoted carbon and nitrogen cycling during composting.

## Conclusions

The CSL, rich in soluble sugars and proteins, provides energy material and nutrients for microbial activities. And adding CSL to SMS compost could change the bacterial community structure and increase the richness and diversity of the bacterial community. Core and functional bacteria are the main driving factors of carbon and nitrogen transformation during composting. The addition of CSL stimulated the activity of low-abundance bacteria and the compensatory effect of the core and functional bacteria. After adding CSL, the core bacterial genus was divided into two categories, synthetic and degrading bacteria, with more synthetic bacteria than degrading bacteria, while there were only degrading bacteria in CK compost. Adding CSL increased the abundance of core and functional bacteria involved in carbon and nitrogen conversion, thus accelerating the degradation of organic carbon and nitrogen, increasing the carbon and nitrogen nutrient sequestration, and improving the composts' quality. Overall, these results provide a theoretical basis for accelerating material transformation during SMS composting.

## Supplementary Information

**Additional file 1:** **Figure** **S1.** Schematic diagram of temperature changes during composting.The figure shows the ambient temperature over the entire composting period and the temperature changes and differences between the two treatments. It is obvious that the temperature of CP treatment is always higher than that of CK control group.

**Additional file 2:** **Figure** **S2.** Schematic diagram of NO3^-^changes during composting.The figure shows the NO3^-^ content and its changing trend in CP and CK treatments during the whole composting period.

**Additional file 3:** **Table** **S1.** Topological features in co-occurrence network of microbial community.

**Additional file 4.** Data.

## Data Availability

All data generated or analyzed during this study are included in the article.
