# Supplementary material for: Effects of adding corn steep liquor on bacterial community composition and carbon and nitrogen transformation during spent mushroom substrate composting
Source: BMC Microbiol. 2023 May 27;23:156. doi: 10.1186/s12866-023-02894-x (PMC10224591; doi:10.1186/s12866-023-02894-x)
Supplement: Supplementary file 1 — Additional file 1: Figure S1. Schematic diagram of temperature changes during composting.The figure shows the ambient temperature over the entire composting period and the temperature changes and differences between the two treatments. It is obvious that the temperature of CP treatment is always higher than that of CK control group. [file 12866_2023_2894_MOESM1_ESM.docx]

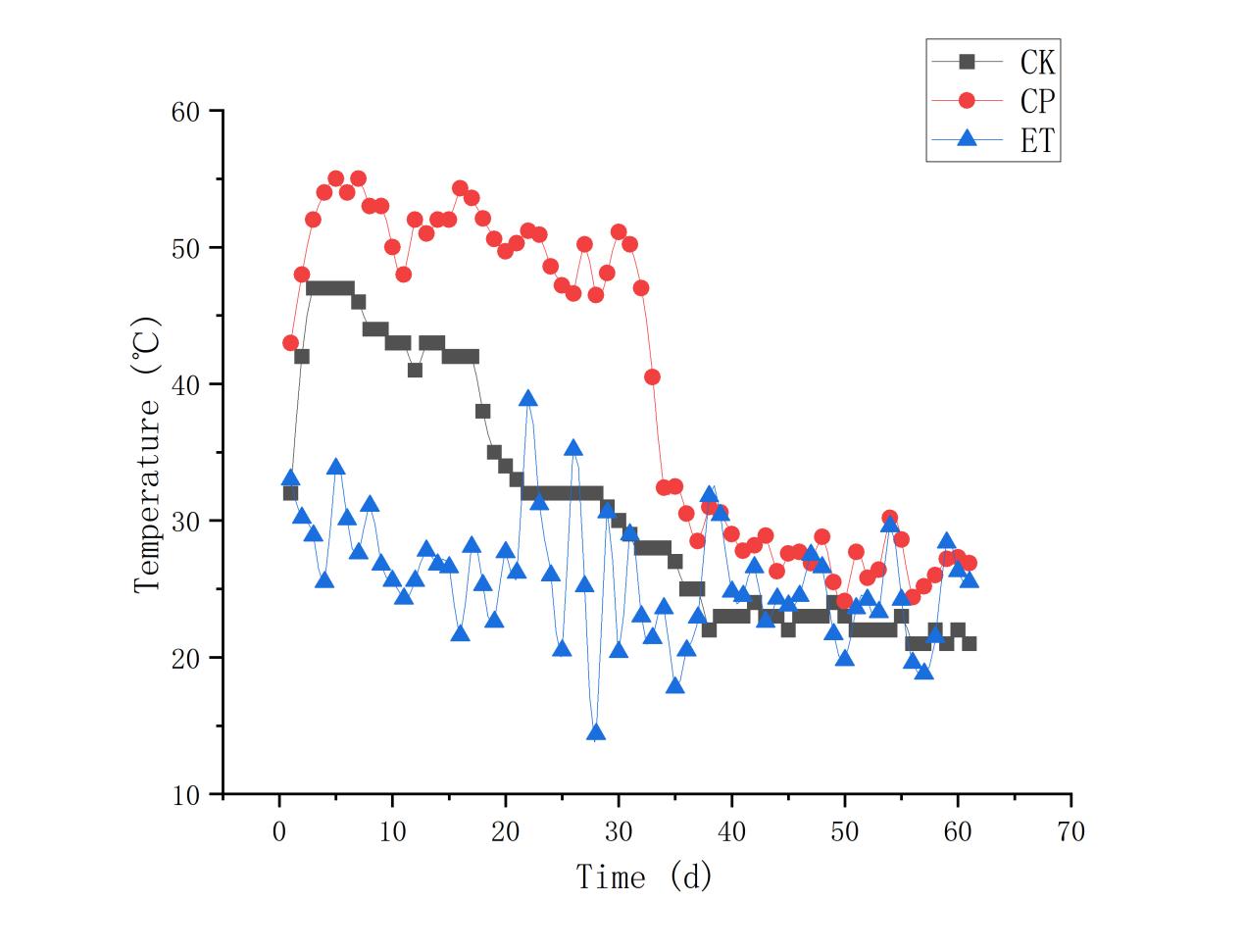


**Figure S1.** Schematic diagram of temperature changes during composting.The figure shows the ambient temperature over the entire composting period and the temperature changes and differences between the two treatments. It is obvious that the temperature of CP treatment is always higher than that of CK control group.
