# Supplementary material for: Effects of adding corn steep liquor on bacterial community composition and carbon and nitrogen transformation during spent mushroom substrate composting
Source: BMC Microbiol. 2023 May 27;23:156. doi: 10.1186/s12866-023-02894-x (PMC10224591; doi:10.1186/s12866-023-02894-x)
Supplement: Supplementary file 2 — Additional file 2: Figure S2. Schematic diagram of NO3-changes during composting.The figure shows the NO3- content and its changing trend in CP and CK treatments during the whole composting period. [file 12866_2023_2894_MOESM2_ESM.docx]

**
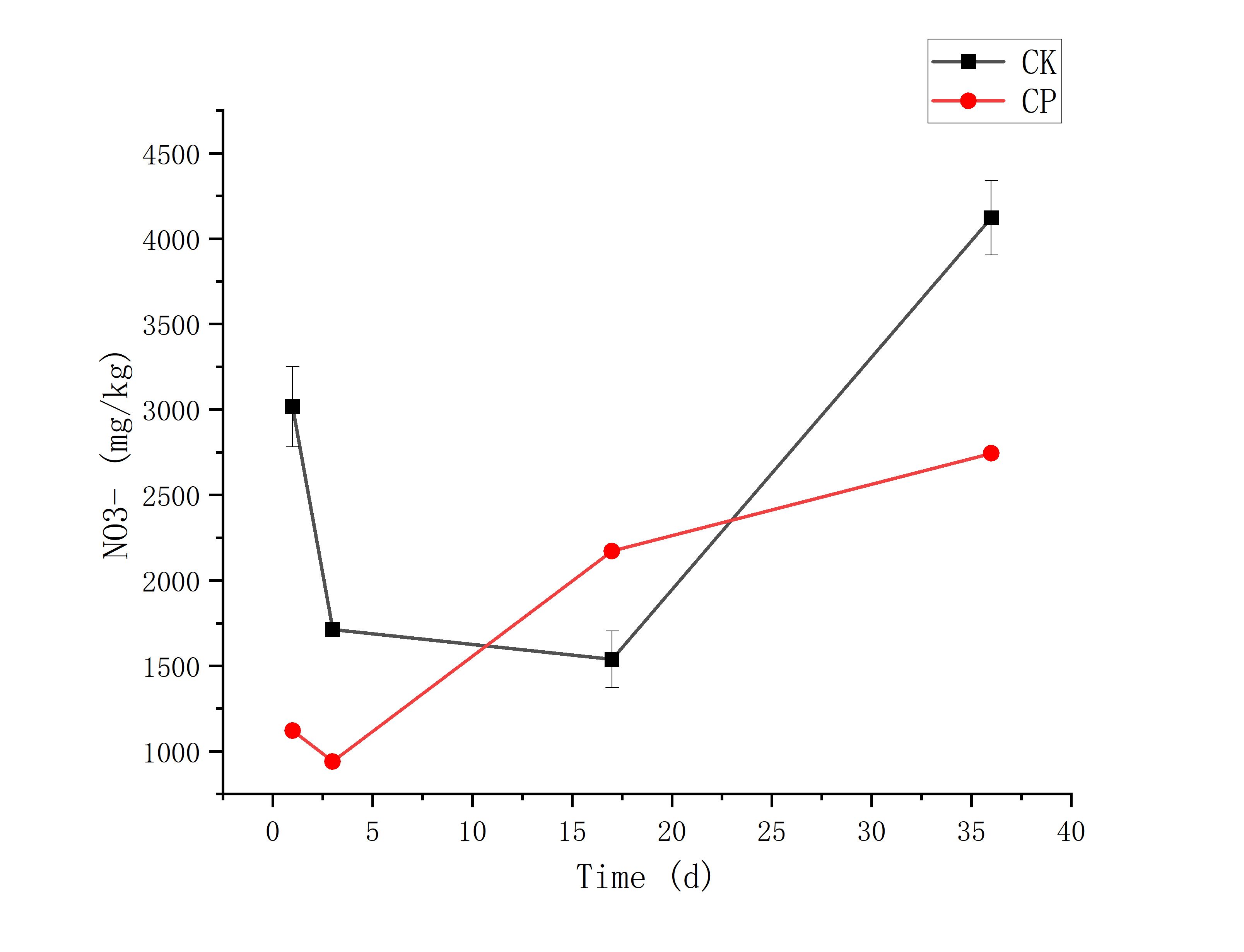
Figure S2.** Schematic diagram of NO3^-^changes during composting.The figure shows the NO3^-^ content and its changing trend in CP and CK treatments during the whole composting period.
