# Supplementary material for: Effects of adding corn steep liquor on bacterial community composition and carbon and nitrogen transformation during spent mushroom substrate composting
Source: BMC Microbiol. 2023 May 27;23:156. doi: 10.1186/s12866-023-02894-x (PMC10224591; doi:10.1186/s12866-023-02894-x)
Supplement: Supplementary file 3 — Additional file 3: Table S1. Topological features in co-occurrence network of microbial community. [file 12866_2023_2894_MOESM3_ESM.docx]

**Table S1** Topological features in co-occurrence network of microbial community.

| Network indexes | CK | CP |
| --- | --- | --- |
| Nodes | 207 | 172 |
| Edges | 625 | 246 |
| Total triangles | 1412 | 158 |
| Average Degree | 6.039 | 2.86 |
| Density | 0.029 | 0.017 |
| Network Diameter | 13 | 15 |
| Average Path length | 4.608 | 4.742 |
| Average Clustering Coefficient | 0.652 | 0.518 |
| Number of Weakly Connected Components | 16 | 28 |
| Number of Communities | 24 | 34 |
| Modularity | 0.651 | 0.813 |
